# Selection, Characterization and Application of Artificial DNA Aptamer Containing Appended Bases with Sub-nanomolar Affinity for a Salivary Biomarker

**DOI:** 10.1038/srep42716

**Published:** 2017-03-03

**Authors:** Hirotaka Minagawa, Kentaro Onodera, Hiroto Fujita, Taiichi Sakamoto, Joe Akitomi, Naoto Kaneko, Ikuo Shiratori, Masayasu Kuwahara, Katsunori Horii, Iwao Waga

**Affiliations:** 1 Innovation Laboratory, NEC Solution Innovators, Ltd., 1-18-7, Shinkiba, Koto-Ku, Tokyo 136-8627, Japan; 2 Graduate School of Science and Technology, Gunma University, 1-5-1 Tenjin-cho, Kiryu, Gunma 376-8515, Japan; 3 Department of Life and Environmental Sciences, Chiba Institute of Technology, 2-17-1 Tsudanuma, Narashino 275-0016, Japan

## Abstract

We have attained a chemically modified DNA aptamer against salivary α-amylase (sAA), which attracts researchers’ attention as a useful biomarker for assessing human psychobiological and social behavioural processes, although high affinity aptamers have not been isolated from a random natural DNA library to date. For the selection, we used the base-appended base (BAB) modification, that is, a modified-base DNA library containing (*E*)-5-(2-(*N*-(2-(N6-adeninyl)ethyl))carbamylvinyl)-uracil in place of thymine. After eight rounds of selection, a 75 mer aptamer, AMYm1, which binds to sAA with extremely high affinity (*K*_d_ < 1 nM), was isolated. Furthermore, we have successfully determined the 36-mer minimum fragment, AMYm1-3, which retains target binding activity comparable to the full-length AMYm1, by surface plasmon resonance assays. Nuclear magnetic resonance spectral analysis indicated that the minimum fragment forms a specific stable conformation, whereas the predicted secondary structures were suggested to be disordered forms. Thus, DNA libraries with BAB-modifications can achieve more diverse conformations for fitness to various targets compared with natural DNA libraries, which is an important advantage for aptamer development. Furthermore, using AMYm1, a capillary gel electrophoresis assay and lateral flow assay with human saliva were conducted, and its feasibility was demonstrated.

Nucleic acid aptamers are single-stranded DNA or RNA molecules that can tightly and specifically bind to their targets. Since the systematic evolution of ligands by exponential (SELEX) enrichment method was established in 1990^1^,^2^, more than 1,500 aptamers against variously sized targets ranging from small ligands^3^ to cells^4^ have been developed. In respect of their applications as sensing elements for food hygiene, environmental monitoring and clinical diagnosis^5^,^6^, nucleic acids have unique features such as reversible folding^7^,^8^, predictable/programmable self-assembly^9^,^10^, polymerase-catalyzed reproducibility^11^,^12^ and non-enzymatic signal amplification^13^,^14^, which enable fabrications of rapid, sensitive and simple target detection systems^15^,^16^ with bound–free separations^17^. Therefore, aptamer development is still drawing considerable attention, although antibodies for various targets are available at present.

In general, in SELEX methods, aptamer selections are conducted using random DNA or RNA libraries. However, the acquisition of high-affinity aptamers for some targets is known to be difficult presumably due to the limited functionalities and conformations of natural oligonucleotides. To improve the binding affinity and specificity, the development of DNA/RNA aptamers with base modifications has been attempted since the 1990s^18^,^19^. In earlier studies^20^,^21^,^22^, the introduction of base modifications exhibited marginal effects on both binding affinity and specificity. In the middle of the 2000s, the positive effects of base modifications bearing foreign functionalities on binding specificities were demonstrated by acquisitions of modified DNA aptamers for glycoprotein^23^ and an R-thalidomide derivative^24^, respectively. Significant improvement in binding affinities by base modification was first demonstrated in 2010 by slow off-rate modified aptamers (SOMAmers™), i.e. high-affinity aptamers for various protein targets^25^,^26^,^27^. The SOMAmers comprise modified DNA that contains C5-substituted uracils bearing foreign functionalities, e.g. benzyl groups, naphthyl groups and side chains of proteinogenic amino acids such as tryptophan and valine. Thereafter, in 2013, we successfully obtained high-affinity DNA aptamers with BAB modification for a camptothecin derivative^28^, demonstrating for the first time that introduction of intrinsic functionality, i.e. an adenine base, can also lead to the enhancement of target binding affinities. This means that the introduced substituents need not necessarily be foreign functionalities in order to improve aptamer performance.

Saliva is an ideal biological sample that can allow clinicians to non-invasively monitor biomarkers for healthcare^29^,^30^,^31^. Salivary α-amylase (sAA) that digests starch is known as a stress biomarker, in addition to secretory immunoglobulin A and chromogranin A. Direct sympathetic nervous activity and norepinephrine released during stress increase sAA serection^32^,^33^. Furthermore, it was recently discovered that sAA levels are greater in diabetic patients than in non-diabetic patients^34^.

Accordingly, in this study, we conducted the selection of sAA-binding DNA aptamers with and without the BAB modification and attempted to identify the minimum length and modification with target binding activity. Furthermore, we addressed the effects of the introduced functionalities on oligonucleotide folding and examined the feasibility of the obtained aptamers.

## Results and Discussion

### Selection of sAA-binding aptamers

**Figure 1:**
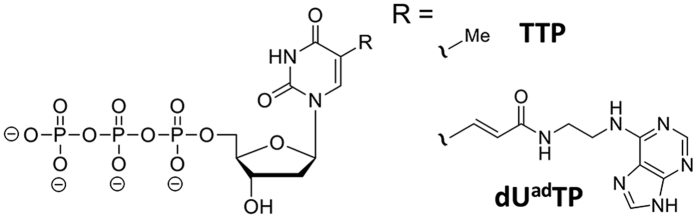
Chemical structures of natural TTP and dU^ad^TP.

Initially, we attempted the acquisition of sAA-binding aptamers three times using a natural single-stranded DNA (ssDNA) library containing a 30-mer random sequence flanked by two primer regions at both ends. Consequently, sAA-binding aptamers were not obtained from the natural ssDNA library after eight rounds of selection. We then conducted the aptamer selection from the modified DNA library enzymatically synthesized using (*E*)-5-(2-(*N*-(2-(N6-adeninyl)ethyl))carbamylvinyl)-2′-deoxyuridine-5′-triphosphate (dU^ad^TP) instead of thymidine-5′-triphosphate (TTP) (Fig. 1). After eight rounds of selection, sequence data of the enriched library were analysed using a next-generation sequencer. Seven sequences, each of which occupies more than 5% of the enriched library, were chosen as aptamer candidates from among determined sequences (Table S1).

**Figure 2:**
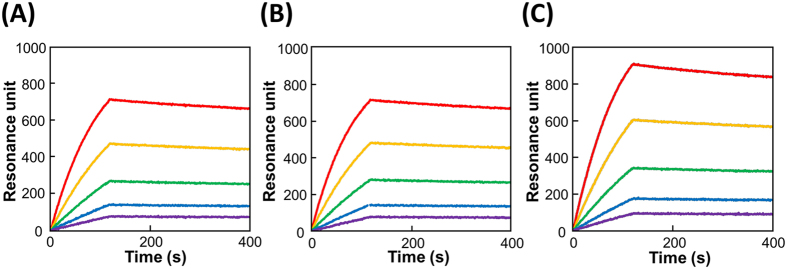
Representative SPR sensorgrams showing the interaction between the target protein sAA and the aptamer (**A**) AMYm1, (**B**) AMYm1-2 or (**C**) AMYm1-3. Measurements were performed using the ProteON XPR360 instrument with multi-cycle kinetics, and various concentrations of sAA (1.25–20 nM) were injected over the respective aptamer-immobilizing sensor chip for 120 s at a flow rate of 50 μL/min.

The binding affinities of these candidates for sAA were measured on a surface plasmon resonance (SPR) instrument. The SPR response signals of the binding event were observed in six candidates when the analyte sAA (400 nM) was injected. The SPR sensorgram of the sAA-binding aptamer with the highest affinity, i.e., AMYm1, is shown in Fig. 2. The dissociation constant (*K*_d_) of AMYm1 binding to the target was determined to be 559 pM (Tables 1 and 2), which is sufficient affinity for practical use as a sensor element.

We then assessed the binding specificity of AMYm1 by SPR measurements using other stress biomarkers in saliva such as secretory immunoglobulin A and chromogranin, instead of sAA. Only small SPR response signals that are nearly comparable to the background level were observed for those non-target proteins.

### Effects of length and modification on binding activity

**Figure 3:**
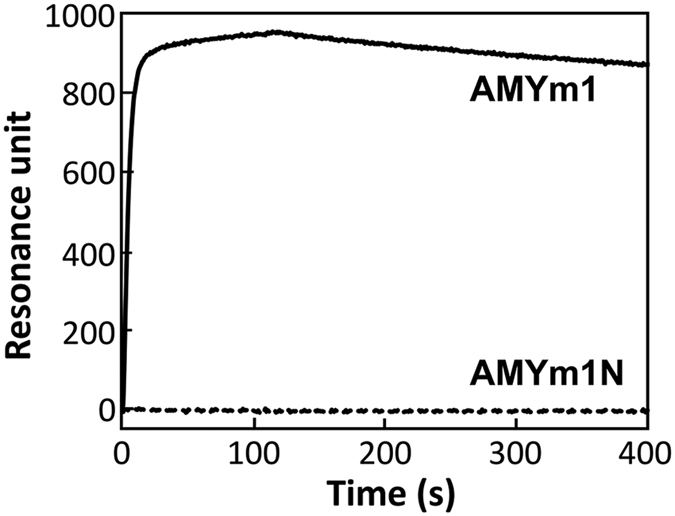
Representative SPR sensorgrams showing the interaction between the target protein sAA and the aptamer AMYm1 or AMYm1N. Measurements were performed using the ProteON XPR360 instrument, and sAA (400 nM) was injected over the respective aptamer-immobilizing sensor chips for 120 s at a flow rate of 50 μL/min.

The best aptamer AMYm1 was found to contain 11 (*E*)-5-(2-(*N*-(2-(N6-adeninyl)ethyl))carbamylvinyl)-uracil (U^ad^) bases in the 30-mer random region. The SPR data showed that the U^ad^-containing ssDNA library did not bind to sAA, indicating that the sequence identified is essential for exerting the target binding activity. In addition, the BAB modification was also found to be essential; the DNA strand in which all U^ad^’s in AMYm1 were replaced with natural thymine, i.e. AMYm1N, did not exhibit target binding activity (Table 1, Fig. 3). As shown in Table 2, 50-mer AMYm1-2 and 36-mer AMYm1-3 retained sub-nanomolar affinity to the target, whereas 32-mer AMYm1-4, which is only four nucleotides shorter than AMYm1-3, lost the binding activity. Four fragments with sequences and lengths identical to the minimum aptamer AMYm1-3, in which a few U^ad^ residues are replaced with natural T, i.e. AMYm1-3-1, AMYm1-3-2, AMYm1-3-3 and AMYm1-3-4, exhibited little or no binding affinity to the target (Table 1 and Figure S1). The results indicate that the introduced substituents play an important role in sustaining the active conformation as an sAA binder.

### NMR analysis of the minimized aptamer

**Figure 4:**
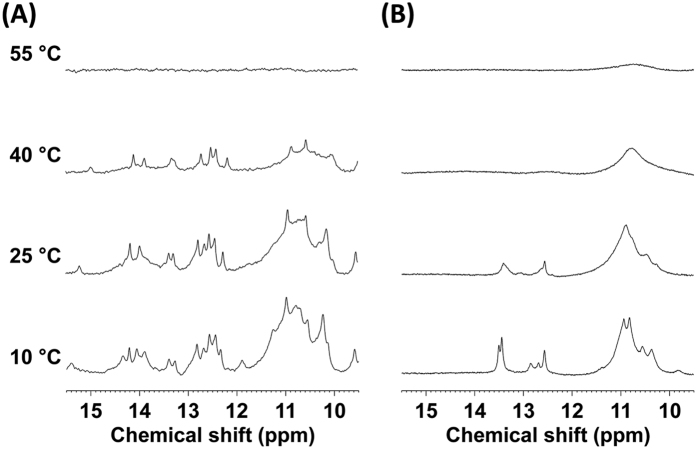
Imino proton spectra of (**A**) the sAA aptamer AMYm1-3 and (**B**) AMYm1-3N, the one without modifications, recorded at different temperatures between 10 °C and 55 °C.

To elucidate the structural features of the sAA-binding aptamer, imino proton spectra of the minimized aptamer AMYm1-3 and the one without modifications AMYm1-3N were measured at various temperatures (Fig. 4). Although imino proton signals could not be assigned because of overlap and broadening, typical imino proton signals of canonical Watson–Crick base pairs were observed at 12–15 ppm. Furthermore, signals at 10–11.5 ppm could be assigned to the imino protons of non-canonical base pairs and the H9N9 imino protons of the adenine part of U^ad^. Exchangeable imino signals can be observed when imino protons are involved in hydrogen bonding or are protected from exchange with the bulk solvent water. Thus, AMYm1-3 may adopt a defined structure in which the imino protons are protected from exchange and is stable at 40 °C.

While a stem-loop structure could be predicted based on the secondary structure of AMYm1-3N using the Zuker Mfold^35^ (Figure S2), several peaks were actually observed at 12–15 ppm in the NMR spectra of AMYm1-3N at 10 °C and 25 °C. As mentioned above, the NMR spectra of AMYm1-3 indicated that the H9N9 imino protons of the adenine part of U^ad^ may be involved in the extra hydrogen-bonding interactions, potentially stabilizing the folded aptamer structure. The variable temperature UV absorption spectra showed that the melting temperature (T_m_) of AMYm1-3 was higher than that of AMYm1-3N (i.e. 44.9 °C and 40.6 °C, respectively; Figure S3). In addition, the circular dichroism (CD) spectra analysis supports the possibility of distinct structure folding of AMYm1-3, which can be induced by the BAB modification, from the folding of AMYm1-3N (Figure S4). Similarly, structural analyses of SOMAmers suggested that the introduced foreign aromatic functionalities imparted thermodynamic stability to the folded structure, which is not achievable with natural DNA^27^.

### Detection of sAA in human saliva

**Figure 5:**
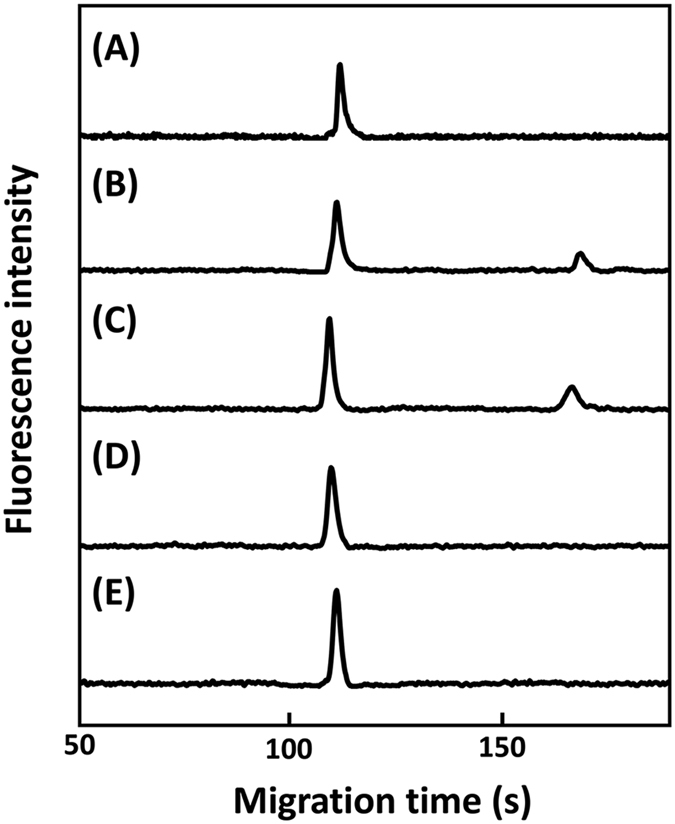
Electropherograms of (**A**) 200 nM AMYm1, (**B**) 200 nM AMYm1 with 2 μM sAA, (**C**) 200 nM AMYm1 with 10% human saliva, (**D**) 200 nM random oligo with 2 μM sAA and (**E**) 200 nM random oligo with 10% human saliva. The aptamer AMYm1 and the random oligo were both labelled with TYE665 at their 5′-end.

To verify the usability of the aptamer AMYm1 for detection of sAA in human saliva, electrophoretic mobility shift assays were performed according to the reported capillary gel electrophoresis method^36^. The aptamer and the DNA library (i.e. random oligo) with the length of 75-mer, which were labelled with a fluorescent dye (TYE665) at the 5′-end, were used. Capillary electrophoreses of the TYE665-labelled AMYm1 and the random oligo with 10% human saliva as well as with sAA (200 nM) as a control were performed (Fig. 5). When the mixture of AMYm1 and sAA was applied, the AMYm1/sAA complex migrated slower than free AMYm1; the peaks of the free aptamer and the complex appeared around 120 and 240 s, respectively (Fig. 5B). The complex peak was clearly observed with the mixture of AMYm1 and 10% human saliva (Fig. 5C). However, no complex peak was observed when the random oligo was used instead of the aptamer (Fig. 5E), showing that the aptamer acts as a specific binder of sAA in human saliva.

The high specificity of AMYm1 was also displayed by a pull-down assay sodium dodecyl sulphate–polyacrylamide gel electrophoresis gel (Figure S5), which clearly showed the band of sAA (55 kDa) in addition to extra bands of some proteins in saliva (12, 80 and 90 kDa) that associate with the primer-immobilizing magnetic beads as a control. The band which appeared at 55 kDa was confirmed to be human sAA by tandem mass spectrometry analysis.

AMYm1, AMYm1-2 and AMYm1-3 exhibited low binding affinity to pancreatic α-amylase (pAA); the *K*_d_ value of pAA was approximately 2.5–5.5 times higher than that of sAA (Table S2). Such specificities may not be sufficiently precise to distinguish between cognate α-amylases in the blood; however, they should be sufficient to detect secreted sAA in the saliva.

### Verification of test strips using an sAA-binding aptamer

**Figure 6:**
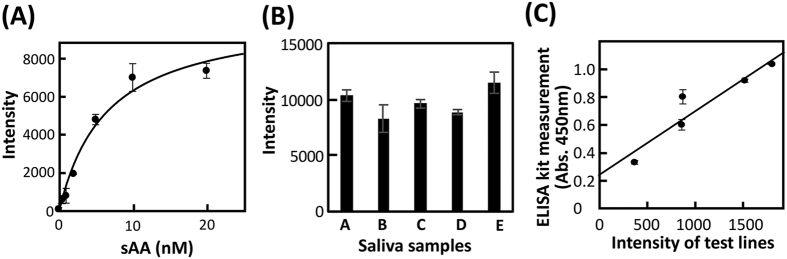
Lateral flow assays using the aptamer–GNP conjugates. (**A**) Calibration curve for sAA using the lateral flow device. The intensities of the test line for each concentration of sAA were determined in duplicate. The line was drawn using a non-linear least-squares fit. (**B**) Assay of 0.1% human saliva samples derived from five people (**A**–**E**). The intensities of the test line were determined in triplicate, and the error bar represents the standard deviation. (**C**) Correlation between the lateral flow and ELISA assays for different concentrations of sAA in 0.05% human saliva. The intensities of the test lines were subtracted from the background level, which was measured in an area near the test line.

Using AMYm1, we prepared test strips for a lateral flow assay based on a sandwich assay method (Figure S6). The target protein sAA and anti-sAA antibody as a capture antibody, which binds to a part of the sAA that differs from the AMYm1 recognition site, were immobilized on the control line and the test line, respectively. With an analyte sample, AMYm1-immobilizing gold nanoparticles (GNPs) were applied on the test strips. The colour on the test line intensified as sAA concentration increased (Fig. 6A, Figure S7). Then, the test strips were verified with 0.1% human saliva (n = 5) instead of sAA, and the test lines exhibited positive results for all five examined samples (Fig. 6B, Figure S8). The correlation between outcomes using the test strips and the human α-amylase enzyme-linked immunosorbent assay (ELISA) kit was verified. As shown in Fig. 6C, high correlation was found between these two assay methods, indicating that AMYm1 can facilitate the quantitative detection of sAA in human saliva.

## Conclusions

Several trials for sAA-binding aptamer acquisition from a natural DNA library have been unsuccessful. However, by using a base-modified DNA library, we have successfully obtained a DNA aptamer with the BAB modification, AMYm1, that exhibits sub-nanomolar affinity to the target protein sAA. The minimized aptamer, a 36-mer of AMYm1-3, retained target binding activity comparable to the full-length AMYm1. Intriguingly, in addition to the BAB modification, the four nucleotides, which include a single U^ad^ residue, at the 5′-end of AMYm1-3 were proven to be critical for the target binding by SPR analyses. NMR analyses of AMYm1-3 suggested that the introduced functionality can contribute to form an unusual defined conformation. The results indicate that BAB modifications can expand the conformational diversity of single-stranded oligonucleotides, leading to the improvement of target binding affinity and specificity. The usability of AMYm1 for detection of ssAA in human saliva was demonstrated by capillary electrophoresis analysis, a pull-down assay and a lateral flow assay. We are currently expanding the repertoire of BAB modifications and developing high-affinity modified DNA aptamers for various biomarkers and drug targets.

## Experimental Procedure

### Materials

The target protein, sAA, was purchased from Lee BioSolutions Inc. (Maryland Heights, MO, USA). Dynabeads M-270 tosyl-activated magnetic beads and Dynabeads MyOne SA C1 magnetic beads were purchased from Invitrogen (Carlsbad, CA, USA). *KOD Dash* DNA polymerase was purchased from Toyobo Co., Ltd. (Osaka, Japan), and synthetic deoxyribooligonucleotides were purchased from Integrated DNA Technologies MBL K.K. (Tokyo, Japan) and Japan BioService (Saitama, Japan). Methyl cellulose and streptavidin were obtained from Sigma-Aldrich Co. LLC (St. Louis, MO, USA), and 40-nm GNPs were obtained from BBI Solutions (Cardiff, UK). For the lateral flow device, the Hi-Flow Plus HF120 membrane was purchased from Millipore (Billerica, MA, USA), and the CF7 absorption pad was purchased from GE Healthcare (Little Chalfont, UK). The antibody, anti-human salivary amylase clone 3F9 mAb, was purchased from Cell Sciences, Inc. (Canton, MA, USA). Human amylase AssayMax ELISA Kit was purchased from Assaypro, LLC (St. Charles, MO, USA) to measure the volume of amylase secreted in human saliva. All other reagents used were of research grade.

### Human saliva

Methods were conducted according to the relevant guidelines. Informed consent was obtained from all the participants. All experiments on the use of human saliva were approved by the Institutional Biosafety Committee of NEC Solution Innovators, Ltd.

### SELEX procedures

The amplification and replication of oligonucleotide strands selected by affinity separation with their target are key processes in the SELEX procedure. In particular, when modified DNA/RNA is used as a library^18^,^19^,^20^,^21^,^22^,^23^,^24^,^25^,^26^,^27^,^28^,^37^,^38^,^39^,^40^, the success or failure of the selection can greatly depend on the polymerase used, which affects the accuracy of modified nucleotide incorporation and the efficiency of modified oligonucleotides production^41^,^42^,^43^,^44^,^45^. In 2001, Sawai *et al*. first discovered that *KOD Dash* DNA polymerase is one of the most promising candidates as a catalyst for enzymatic syntheses of modified DNAs^37^,^46^,^47^,^48^,^49^,^50^. Thereafter, *KOD* DNA polymerase-related products (*e.g. KOD XL, KOD FX, AccuPrime Pfx* and *KOD Dash)* have been widely employed in applications for enzymatic syntheses of modified DNAs involving SELEX selections using modified oligonucleotide libraries^26^,^28^,^51^,^52^,^53^. Also, in this study, we used *KOD Dash* DNA polymerase for aptamer selections.

The target protein, sAA, was immobilized on Dynabeads M-270 tosyl-activated magnetic beads according to the manufacturer’s instructions. The base-modified nucleoside triphosphate dU^ad^TP was synthesized according to our previously reported method^26^. The initial U^ad^-containing ssDNA library was enzymatically prepared by a primer extension reaction at 70 °C for 30 min using *KOD Dash* DNA polymerase, 2 nmol of the 5′-biotinylated DNA template (5′-GAT TTC GAG ACG GGT GCA ACT C-N_30_-C AAT AGG CGG CGT TAA GGT ATC C-3′), 2.4 nmol of the forward (Fw) primer (5′-GGA TAC CTT AAC GCC GCC TAT TG-3′) and 125 nmol each of four triphosphates (dU^ad^TP, dCTP, dATP and dGTP). To remove the 5′-biotinylated DNA template, the reaction mixture was incubated with 10 mg of Dynabeads MyOne SA C1 magnetic beads. Subsequently, the ssDNA containing U^ad^′s was eluted with 20 mM NaOH from the beads, and the elution was neutralized with 80 mM HCl to yield the initial U^ad^-containing ssDNA library.

We modified the standard SELEX selection protocol^26^ for the sAA-binding aptamer selections using natural and base-modified DNA libraries. Briefly, the natural or modified ssDNA library was mixed with the target beads for 15 min at 25 °C, and then the beads were washed with selection buffer (pH 7.5) containing 40 mM 4-(2-hydroxyethyl)-1-piperazineethanesulfonic acid (HEPES), 125 mM NaCl, 5 mM KCl, 1 mM MgCl_2_, and 0.01% Tween 20. Then, the ssDNA bound to the beads was eluted with 7 M urea, and amplified by the polymerase chain reaction (PCR) using the Fw primer and the 5′-biotinylated reverse (Rv) primer (5′-GAT TTC GAG ACG GGT GCA ACT C-3′). The amplified dsDNA was incubated with Dynabeads MyOne SA C1 magnetic beads, treated with 20 mM NaOH to elute the Fw primer-elongated products, which were use as the natural ssDNA library for the next round, from the beads, and then the elution was neutralized. Meanwhile, preparation of the modified ssDNA library for the next round was performed as follows. The magnetic beads immobilizing the 5′-biotinylated Rv primer-elongated products, which were obtained by removal of Fw primer-elongated products after the PCR amplification of strands selected by the abovementioned affinity separation, were mixed and incubated with the Fw primer, triphosphates (dU^ad^TP, dCTP, dATP, and dGTP) and *KOD Dash* DNA polymerase. The Fw primer-elongated products were eluted with 20 mM NaOH, and the elution was neutralized.

After the enrichment of the active species was confirmed, the enriched library was amplified by PCR using non-labelled Fw and Rv primers and then sequenced with a GS junior sequencer (Roche, Indianapolis, IN, USA). Detailed protocols of sample preparation for next-generation sequencing (NGS) are provided in the supplementary information. The 10 most common sequences from the natural and modified ssDNA libraries are listed in Tables S3 and S4, respectively.

### SPR assays

The SPR assay was performed at 25 °C using the ProteON XPR360 instrument (Bio-Rad Laboratories, Inc., Hercules, CA, USA). A 20-mer deoxyadenylic homooligonucleotide was attached to the 3′-end of an aptamer, which was hybridized with a 5′-biotinylated 20-mer thymidylic homooligonucleotide immobilized onto a NLC sensor chip (NeutrAvidin)^4^. The target protein, sAA, was used as the analyte, and the abovementioned selection buffer (pH 7.5) was used as the running buffer. To analyse binding specificities, pAA was used instead of sAA. The association and dissociation rate constants (*k*_a_ and *k*_d_, respectively) and the dissociation constants (*K*_d_ values) were determined using a global fitting analysis with a simple Langmuir model of 1:1 binding, based on the assumption that the immobilized aptamers bind to the analyte in a 1:1 ratio.

### NMR measurements

The minimized aptamer AMYm1-3 and the one without modifications AMYm1-3N were annealed by heating at 95 °C for 5 min, followed by snap-cooling on ice. The sample was dissolved in 10 mM sodium phosphate buffer (pH 6.5). The final concentration of the sample was 0.4 mM. NMR spectra were collected using an AVANCE-600 spectrometer (Bruker BioSpin GmbH, Rheinstetten, Germany) at probe temperatures of 10 °C, 25 °C, 40 °C and 55 °C. The one-dimensional imino proton spectra were recorded using the jump-and-return scheme for water suppression^54^.

### Capillary gel electrophoresis assays

The TYE665-labelled aptamer or random oligo (10 μL, 200 nM) was incubated for 30 min at 25 °C with or without sAA (2 μM) or 10% human saliva in 10 μL of buffer solution (pH 7.5): 40 mM HEPES, 125 mM NaCl, 5 mM KCl and 1 mM MgCl_2_. Electrophoresis was performed on a 0.6% methyl cellulose gel using a microchip electrophoresis system, COSMOEYE SV1210 (Hitachi High-Technologies Corporation, Tokyo, Japan). Human saliva was collected using the Saliva Collection Aid (Salimetrics, LLC, State College, PA, USA).

### Lateral flow assays

GNPs were coated with streptavidin and mixed with the biotinylated aptamer AMYm1 to prepare aptamer–GNP conjugates. The Hi-Flow Plus HF120 membrane and the CF7 absorption pad were used for preparation of the lateral flow test strips^55^. Initially, sAA was applied on the control line, and then the anti-sAA antibody as a capture antibody was applied on the test line using the BioJet Quanti dispenser (BioDot Inc., Irvine, CA, USA), followed by drying for 1 h at 50 °C. The test strips were developed with a mixture of the aptamer–GNP conjugates and 0.1% human saliva in the selection buffer containing 1% bovine serum albumin. Instead of the analyte human saliva, sAA with different concentrations (0.5–20 nM) was used to obtain the calibration curve. The test strips were analysed using a ChemiDoc XRS + imaging system (Bio-Rad Laboratories, Inc.) to quantify the levels of amylase in the human saliva. The amylase levels were also measured using the human amylase AssayMax ELISA Kit to verify the present AMYm1-based detection system. Detailed protocols for preparing aptamer–GNP conjugates and aptamer-based test strips are provided in the supplementary information.

## Additional Information

**How to cite this article:** Minagawa, H. *et al*. Selection, Characterization and Application of Artificial DNA Aptamer Containing Appended Bases with Sub-nanomolar Affinity for a Salivary Biomarker. *Sci. Rep.* **7**, 42716; doi: 10.1038/srep42716 (2017).

**Publisher's note:** Springer Nature remains neutral with regard to jurisdictional claims in published maps and institutional affiliations.

## Supplementary Material

Supplementary Information

## Figures and Tables

**Table 1:** Sequences of the sAA-binding aptamer AMYm1 and their fragments and variants.

Aptamer	Sequence^a^
AMYm1	GGATACCTTAACGCCGCCTATTG**t**GAACGACG**t**GAA**t**AG**t**G**ttt**G**t**GGG**t**CCGGAG**tt**GCACCCG**t**C**t**CGAAA**t**C
AMYm1N	GGATACCTTAACGCCGCCTATTGTGAACGACGTGAATAGTGTTTGTGGGTCCGGAGTTGCACCCGTCTCGAAATC
AMYm1-2	TAACGCCGCCTATTG**t**GAACGACG**t**GAA**t**AG**t**G**ttt**G**t**GGG**t**CCGGAG**tt**
AMYm1-3	G**t**GAACGACG**t**GAA**t**AG**t**G**ttt**G**t**GGG**t**CCGGAG**tt**
AMYm1-3N	GTGAACGACGTGAATAGTGTTTGTGGGTCCGGAGTT
AMYm1-3-1	G**t**GAACGACG**t**GAA**t**AG**t**GTTTGTGGGTCCGGAGTT
AMYm1-3-2	GTGAACGACGTGAATAG**t**G**ttt**G**t**GGG**t**CCGGAG**tt**
AMYm1-3-3	GTGAACGACGTGAA**t**AG**t**G**ttt**G**t**GGG**t**CCGGAG**tt**
AMYm1-3-4	G**t**GAACGACG**t**GAA**t**AG**t**GTTTGTGGGTCCGGAG**tt**
AMYm1-4	ACGACG**t**GAA**t**AG**t**G**ttt**G**t**GGG**t**CCGGAG**tt**

^a^Sequences are aligned in the 5′ to 3′ direction. Underlined regions are derived from the primer or primer-binding regions. Bold letters (**t**) indicate (*E*)-5-(2-(*N*-(2-(*N*^6^-adeninyl)ethyl))carbamylvinyl)-uracil (U^ad^).

**Table 2:** Apparent association and dissociation constants and *K*_d_ values of sAA as determined using SPR assays with the ProteON XPR360.

Aptamer	*k*_*a*_ (M^−1^ · s^−1^)	*k*_*d*_ (s^−1^)	*K*_d_ (pM)
AMYm1	4.16 ± 0.70 × 10^5^	2.30 ± 0.13 × 10^−4^	559 ± 59
AMYm1-2	4.80 ± 0.83 × 10^5^	2.14 ± 0.12 × 10^−4^	451 ± 60
AMYm1-3	3.93 ± 0.98 × 10^5^	2.33 ± 0.22 × 10^−4^	607 ± 88
