# Supplementary material for: Selection, Characterization and Application of Artificial DNA Aptamer Containing Appended Bases with Sub-nanomolar Affinity for a Salivary Biomarker
Source: Sci Rep. 2017 Mar 3;7:42716. doi: 10.1038/srep42716 (PMC5335659; doi:10.1038/srep42716)
Supplement: Supplementary Information [file srep42716-s1.pdf]

Supplementary information

Selection, Characterization and Application of Artificial DNA Aptamer Containing Appended Bases with Sub-nanomolar Affinity for a Salivary Biomarker

Hirotaka Minagawa[1], Kentaro Onodera[2], Hiroto Fujita[2], Taiichi Sakamoto[3], Joe Akitomi[1], Naoto Kaneko[1], Ikuo Shiratori[1], Masayasu Kuwahara[2], Katsunori Horii[1], and Iwao Waga[1]

[1] Innovation Laboratory, NEC Solution Innovators, Ltd., 1-18-7, Shinkiba, Koto-Ku, Tokyo 136-8627, Japan

[2] Graduate School of Science and Technology, Gunma University, 1-5-1 Tenjin-cho, Kiryu, Gunma 376-8515, Japan

[3] Department of Life and Environmental Sciences, Chiba Institute of Technology, 2-17-1 Tsudanuma, Narashino 275-0016, Japan

Correspondence:

Professor M. Kuwahara, Gunma University (E-mail: mkuwa@gunma-u.ac.jp)

Dr. K. Horii, NEC Solution Innovators, Ltd. (E-mail: kat-horii@te.jp.nec.com)

Detailed protocols for experiments other than those covered in the main EXPERIMENTAL PROCEDURE

Sample preparation for NGS

Selection pool DNA after the 8th round was amplified using 2 μM of Fw and unbiotinylated Rv primers, 1.25 U of *KOD Dash* and *KOD Dash* buffer (Toyobo Co., Ltd., Osaka, Japan) and 0.2 mM of dNTPs. The resulting PCR products were purified using NucleoSpin Gel and PCR Clean-up (Macherey-Nagel GmbH & Co., Germany) and were then prepared as samples using the GS FLX Titanium Rapid Library Preparation Kit (Roche, Indianapolis, IN, USA), according to the manufacturer's instructions. Emulsion PCR was performed using GS Junior Titanium emPCR Kit (Roche, Indianapolis, IN, USA), and sample beads were purified using GS Junior Titanium PicoTiterPlate and GS Junior Titanium Sequencing Kits (Roche, Indianapolis, IN, USA), according to the manufacturer's instructions.

Sequence analysis

The 10 most common sequences from NGS are listed in Tables S3 and S4. These sequences accounted for >90% of each DNA library. We set >5% as the frequency threshold for affinity validation.

UV melting curve analysis

The UV melting curves were recorded using V-730BIO (JASCO Co., Tokyo, Japan) with an eight-sample cell changer in quartz cells of 1-cm path length. Variation in the UV absorbance and temperature was monitored at 260 nm. The temperature was measured between 10 °C and 90 °C, and the rate of temperature increase was set to 1 °C/min. The samples (AMYm1-

3 and AMYm1-3N) were dissolved in 10 mM sodium phosphate buffer (pH 6.5). Melting temperature was estimated from the first derivative of the melting curve (Figure S3).

CD analysis

The AMYm1-3 and AMYm1-3N samples were dissolved in 10 mM sodium phosphate buffer (pH 6.5). Then, the CD spectra were measured at 25 °C using a J-725 CD spectrometer (JASCO Corporation, Japan) with a wavelength range of 200–350 nm using a quartz cuvette with a 1.0-mm optical path length (Figure S4). The scanning speed was set to 20 nm/min, and the response time was 16 ms.

Preparation of aptamer–GNP conjugates

GNP solution (2.7 mL) was mixed with 300 μL of streptavidin (SA; 60 μg/mL) at room temperature for at least 10 min. Then, 100 μL of 1% PEG 20,000 (Wako, #168-11285, Japan) and 200 μL of 10% BSA were added to the mixture, which was centrifuged at 8,000 × *g* for 15 min. The supernatant was discarded, and 6 mL of GNP preservation solution (20 mM Tris, 0.05% PEG 20,000, 150 mM NaCl and 1% BSA, pH 8.2) was added to the pellet. The samples were again centrifuged at 8,000 × *g* for 15 min, the supernatant was discarded and 6 mL of GNP preservation solution was added (washing step). SA-coated GNP and 14 μL of 100 μM biotinylated aptamer were mixed at room temperature for 30 min. The aptamer–GNP conjugates were washed as described earlier, and diluted with GNP preservation solution so that the absorbance at 520 nm was 0.60 in a cell of path length 1 mm.

Preparation of aptamer-based test strips

Test strips comprised nitrocellulose membrane (Hi-Flow Plus HF120, Millipore) and absorption pad (CF7, GE Healthcare; Figure S6). The test zone on the nitrocellulose

membrane was prepared using the anti-human salivary amylase antibody solutions (1 mg/mL). We tested three types of antibodies–anti-human salivary amylase clone 2E9, 3F9 and 3C9 mAb (Cell Sciences, Inc.); only 3F9 was found to be useful for sandwich ELISA. The 2E9 and 3C9 antibodies were unusable because they non-specifically bound to aptamer–GNP conjugates, producing a false-positive band on the test zone without the target sAA. The control zone on the nitrocellulose membrane was prepared using sAA (1 mg/mL). The distance between the test and control zones was approximately 0.5 cm. After subsequently dispensing the antibody and sAA, the membrane was air dried at 50°C for 1 h, and the test strips with a 5-mm width were sliced using the cutting module of CM 4000 (BioDot Inc., Irvine, CA, USA).

Table S1. Sequences of the selected sAA-binding aptamers.

Aptamer	Sequence[a]
AMYm1	GGATACCTTAACGCCGCCTATTGtGAACGACGtGAAtAGtGtttGtGGGtCCGGAGttGCACCCGtCtCGAAAtC
AMYm2	GGATACCTTAACGCCGCCTATTGCtAtGAGtAGtGtttGtAGGtCtGGCAtGCGAGttGCACCCGtCtCGAAAtC
AMYm3	GGATACCTTAACGCCGCCTATTGCGAGGtGtGGCtAGtCGttAtAGGtCCACCGAGttGCACCCGtCtCGAAAtC
AMYm4	GGATACCTTAACGCCGCCTATTGAttCtCtGtGACtAGtGtttGtAGGtCGCAGAGttGCACCCGtCtCGAAAtC
AMYm5	GGATACCTTAACGCCGCCTATTGAGttCAACAAtGACtAGtGtttGtCGGtCtGAGttGCACCCGtCtCGAAAtC
AMYm6[b]	GGATACCTTAACGCCGCCTATTGCtCtGtGCCtAttCGAtGtGtCtAtttACtGAGttGCACCCGtCtCGAAAtC
AMYm7	GGATACCTTAACGCCGCCTATTGGtGtCACtAGtGtttGtCGGtGCCAGCAAGGAGttGCACCCGtCtCGAAAtC

[a]Sequences are aligned in the 5′ to 3′ direction. Underlined regions are derived from the primer or primer-binding regions. Bold letters (**t**) indicate (*E*)-5-(2-(*N*-(2-(*N*[6]-adeninyl)ethyl))carbamylvinyl)-uracil (U^{ad}). [b]No binding activity was observed.

Table S2. Apparent association and dissociation constants and K_d values of pAA as determined using SPR assays with the ProteON XPR360.

Aptamer	k_a ($M^{-1} \cdot s^{-1}$)	k_d (s^{-1})	K_d (nM)
AMYm1	2.22×10^5	3.04×10^{-4}	1.37
AMYm1-2	1.74×10^5	4.29×10^{-4}	2.46
AMYm1-3	1.43×10^5	4.29×10^{-4}	3.00

Table S3. The 10 most common sequences from the natural ssDNA library.

No.	Sequence[a]	Frequency
1	GGTAAGACTCCCGCCAGATTTG**AGGCATAACAGGT**CATCTCTGGTGTTATTGTGAAGCCCTCGGTCGAAATC	0.2540
2	GGTAAGACTCCCGCCAGATTTG**AGGCATGTCAGGT**CAAATATGGCGAAACCTGTGAAGCCCTCGGTCGAAATC	0.1662
3	GGTAAGACTCCCGCCAGATTTGA**AGGCATATCAGGT**TTGAATGTGGTGTACAGTGAAGCCCTCGGTCGAAATC	0.1448
4	GGTAAGACTCCCGCCAGATTTGTGACGGCATAACAGGGGCACTAGGCGGGGGTGAAGCCCTCGGTCGAAATC	0.1033
5	GGTAAGACTCCCGCCAGATTTGA**AGGCATGACAGGT**TCAAGCTGGAGATACGGTGAAGCCCTCGGTCGAAATC	0.0962
6	GGTAAGACTCCCGCCAGATTTGA**AGGCATAACAGGT**TTAAATCTGTCAAGCTGTGAAGCCCTCGGTCGAAATC	0.0685
7	GGTAAGACTCCCGCCAGATTTGGGAGGGGGGTGGGGGAGGTGGAGGGGCGGGTGAAGCCCTCGGTCGAAATC	0.0419
8	GGTAAGACTCCCGCCAGATTTG**AGGCATATCAGGT**TAAATATGCGTTGTCGCGTGAAGCCCTCGGTCGAAATC	0.0326
9	GGTAAGACTCCCGCCAGATTTGC**GGCATATCAGG**GCAATTTAGGGAGAGCTCGTGAAGCCCTCGGTCGAAATC	0.0230
10	GGTAAGACTCCCGCCAGATTTGTG**AGGCATATCAGGT**CACATACTCGGGATTGTGAAGCCCTCGGTCGAAATC	0.0155
–	other sequences	0.0540

[a]Sequences are aligned in the 5′ to 3′ direction. Underlined regions are derived from the primer or primer-binding regions. Sequences in boldface are highly conserved, and those highlighted yellow are commonly seen.

Table S4. The 10 most common sequences from the modified ssDNA library.

No.	Sequence[a]	Frequency
1	GGATACCTTAACGCCGCCTATTGTGAACGACG**TGAATAGTGTTTGTGGGTC**CGGAGTTGCACCCGTCTCGAAATC	0.2375
2	GGATACCTTAACGCCGCCTATTGCTA**TGAGTAGTGTTTGTAGGTC**TGGCATGCGAGTTGCACCCGTCTCGAAATC	0.1640
3	GGATACCTTAACGCCGCCTATTGCGAGGTG**TGGCTAGTCGTTATAGGTC**CACCGAGTTGCACCCGTCTCGAAATC	0.1320
4	GGATACCTTAACGCCGCCTATTGATTCTCTG**TGACTAGTGTTTGTAGGTC**GCAGAGTTGCACCCGTCTCGAAATC	0.1256
5	GGATACCTTAACGCCGCCTATTGAGTTCAACAA**TGACTAGTGTTTGTCGGTC**TGAGTTGCACCCGTCTCGAAATC	0.0946
6	GGATACCTTAACGCCGCCTATTGCACGAGTAA**TGACTAGTGTTTGTGGGTC**ACGAGTTGCACCCGTCTCGAAATC	0.0468
7	GGATACCTTAACGCCGCCTATTGA**TGACTAGTGTTTGTCGGTC**GCAAATATGGGAGTTGCACCCGTCTCGAAATC	0.0423
8	GGATACCTTAACGCCGCCTATTGCAACGTGG**TGACTAGTGTTGTTTGGGTC**CAGAGTTGCACCCGTCTCGAAATC	0.0270
9	GGATACCTTAACGCCGCCTATTG**TGATTAGTGTTTGTGGGTC**CAATAGATTAAGAGTTGCACCCGTCTCGAAATC	0.0195
10	GGATACCTTAACGCCGCCTATTGG**TGAATAGTGTTTGTGGGTC**CCTGTCAGGCGAGTTGCACCCGTCTCGAAATC	0.0192
–	other sequences	0.0915

[a]Sequences are aligned in the 5′ to 3′ direction. Underlined regions are derived from the primer or primer-binding regions. Sequences in boldface are highly conserved, and those highlighted yellow are commonly seen.

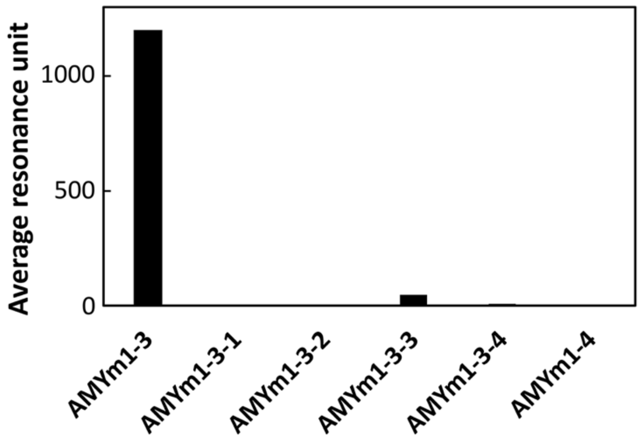

Figure S1. Average SPR response units of the interaction between the target protein sAA and the aptamer (AMYm1-3, AMYm1-3-1, AMYm1-3-2, AMYm1-3-3, AMYm1-3-4, or AMYm1-4). Measurements were performed using the ProteON XPR360, and sAA (400 nM) was injected over the respective aptamer-immobilizing sensor chips for 120 s at a flow rate of 50 μL/min. SPR response units during 115–125 s, which were in the plateau region of the sensorgram curve, were averaged.

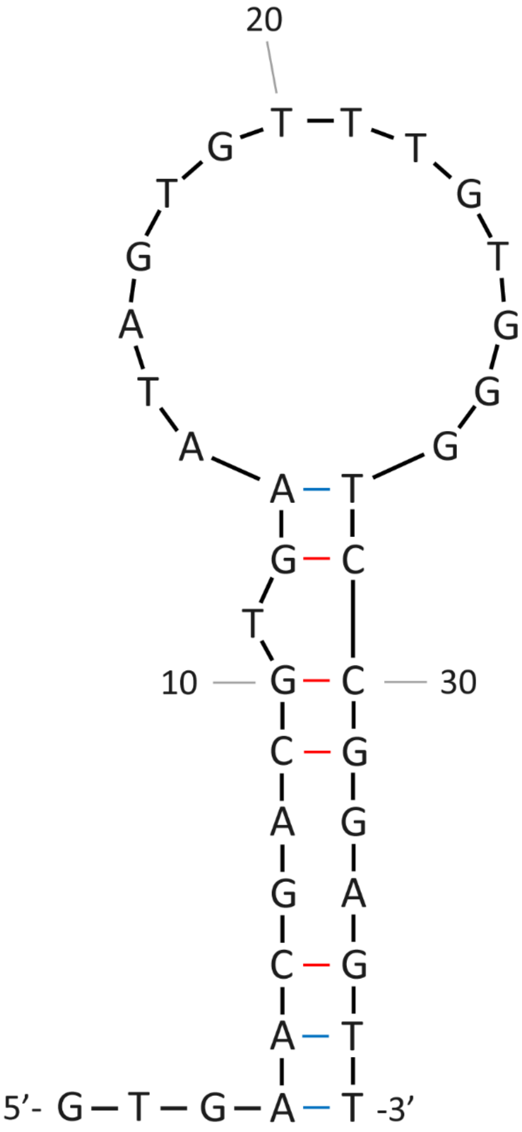

Figure S2. Predicted secondary structure of AMYm1-3N using mfold–DNA folding form.

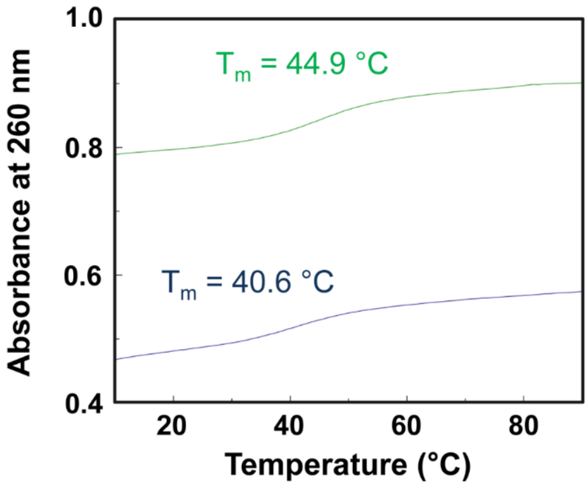

Figure S3. UV melting curve analysis of AMYm1-3 (green) and AMYm1-3N (blue).

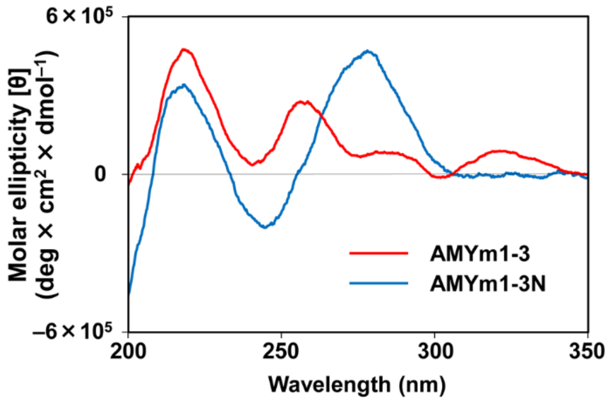

Figure S4. CD spectra for AMYm1-3 (red) and AMYm1-3N (blue).

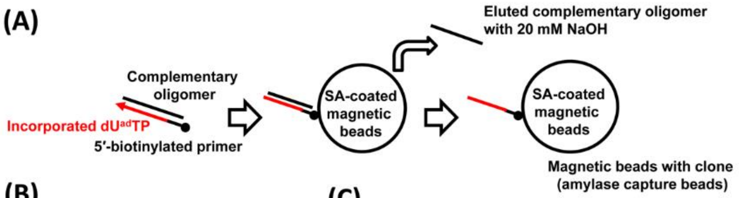

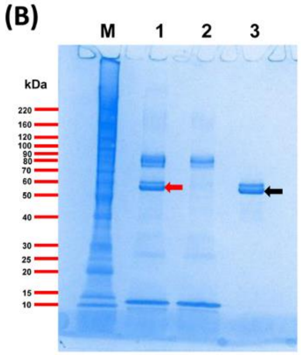

(C)

Identified proteins [specific]	Theoretical mass	Mascot score
α-Amylase [homo sapiens]	58398	1638
Chain A, subsite mapping of the active site of human pancreatic α-amylase using substrates, the pharmacological inhibitor acarbose, and an active site variant [homo sapiens]	56462	1620
BPI fold-containing family B member 2 precursor [homo sapiens]	49256	46
Myeloperoxidase (EC 1.11.1.7), splice form H14-human [homo sapiens]	93405	43

Figure S5. Pull-down assay for human saliva using AMYm1-immobilizing magnetic beads, i.e. sAA capture beads, and primer-immobilizing magnetic beads, i.e. control beads. (A) Schematic illustration of the pull-down assay: 100 μg of the sAA capture beads or the control beads were mixed with human saliva in the selection buffer, incubated at 25°C for 30 min and washed three times with the selection buffer. The bound proteins were eluted with 0.1% SDS at 95°C for 10 min. The eluted samples were electrophoresed on PAGEL C520L (ATTO, Japan), according to the instruction manual. (B) SDS-PAGE of the samples from the pull-down assay: the Bench Mark Protein Ladder molecular size marker (Invitrogen, Carlsbad, CA, USA) (lane M), the sample eluted from the sAA capture beads (lane 1), the sample eluted from the control beads (lane 2) and sAA only (lane 3). The band indicated with the red arrow was analysed by liquid chromatography ion trap time-of-flight mass spectrometry (LCMS-IT-TOF) at Shimadzu Techno-Research, Inc. (Japan). (C) Results of LCMS-IT-TOF analyses: human α-amylase was identified by a Mascot score of 1638.

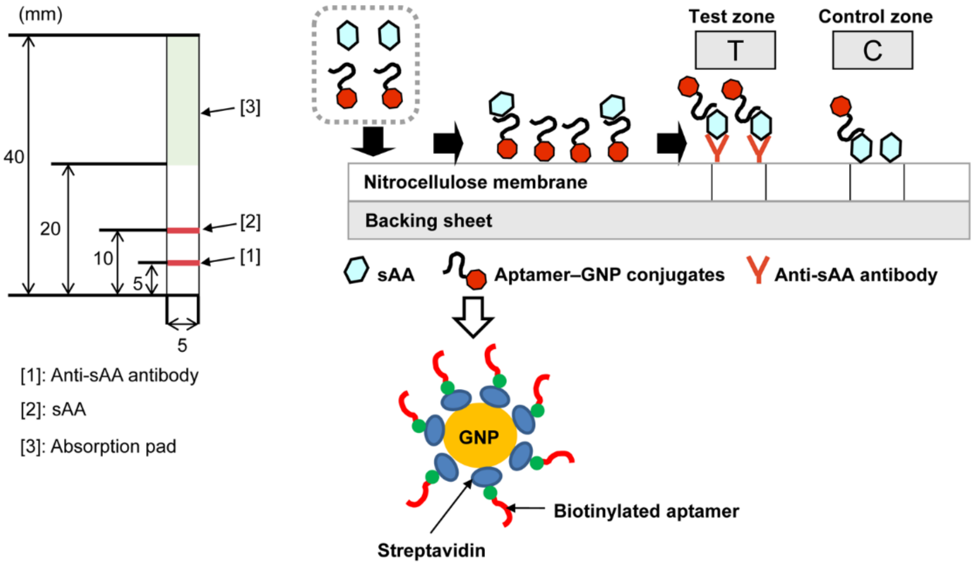

Figure S6. Illustration for the aptamer-based test strips.

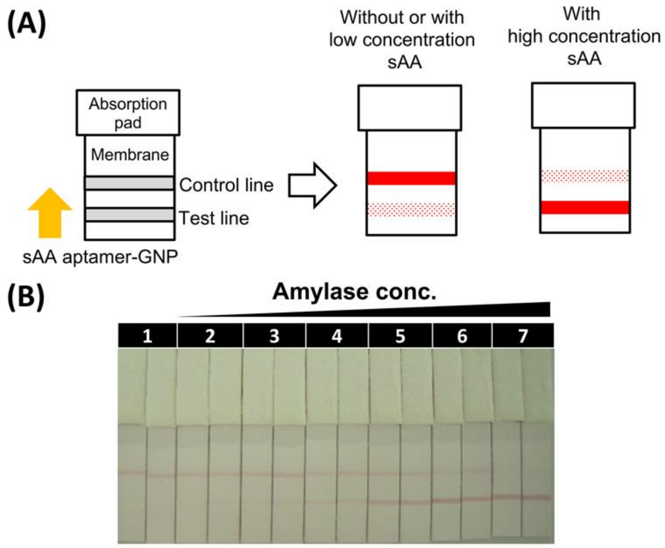

Figure S7. Sandwich binding assay using the aptamer–GNP-based lateral flow device. (A) Schematic illustration of the lateral flow device: the control and test lines on the membrane were prepared by dispensing sAA and the sAA antibody, respectively. If a sample contains sAA, sAA binds to the aptamer–GNP conjugates and is captured by the sAA antibody on the test lines, resulting in an intensified colour of the test line. (B) Test strips 1–7 were developed with samples containing the analyte sAA at concentrations of 0, 0.5, 1, 2, 5, 10 and 20 nM, respectively.

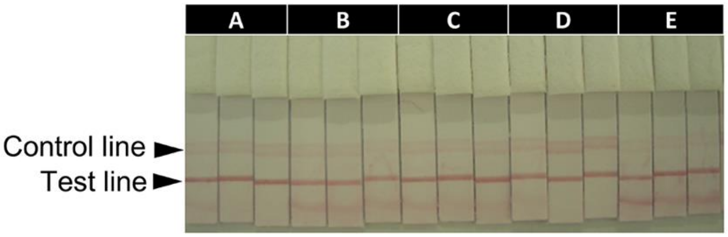

Figure S8. Sandwich binding assay of 0.1% human saliva samples from five people (A–E). The measurements were performed in triplicate.
